# Krüppel-like zinc finger proteins in end-stage COPD lungs with and without severe alpha1-antitrypsin deficiency

**DOI:** 10.1186/1750-1172-7-29

**Published:** 2012-05-23

**Authors:** A-Rembert Koczulla, Danny Jonigk, Thomas Wolf, Christian Herr, Sarah Noeske, Walter Klepetko, Claus Vogelmeier, Nils von Neuhoff, Johanna Rische, Sabine Wrenger, Heiko Golpon, Robert Voswinckel, Maurizio Luisetti, Ilaria Ferrarotti, Tobias Welte, Sabina Janciauskiene

**Affiliations:** 1 Institute of Pathology, Hannover Medical School, Hanover, Germany; 2 Department of Internal Medicine, Division of Pulmonary Diseases, Hospital of the Philipps-Universität Marburg, Baldingerstrasse 1, Marburg, D-35043, Germany; 3 Institute of Molecular Pathology, Hannover Medical School, Hanover, Germany; 4 Department of Theoretical Bioinformatics, German Cancer Research Center DKFZ, Heidelberg, Germany; 5 Department of Internal Medicine V, Pulmonology, Allergology, Respiratory and Environmental Medicine, Saarland University Hospital, Homburg/Saar, Germany; 6 Division of Thoracic Surgery, Department of Surgery, Medical University of Vienna, Vienna, Austria; 7 Department of Respiratory Medicine, Hannover Medical School, Feodor-Lynen-Str. 23, 30625, Hannover, Germany; 8 University of Giessen Lung Center, Department of Internal Medicine, University Hospital Giessen, Giessen, Germany; 9 Center for Diagnosis of Inherited AAT Deficiency, Laboratory of Biochemistry and Genetics, Institute for Respiratory Disease, IRCCS Policlinico San Matteo Foundation, University of Pavia, Pavia, Italy

**Keywords:** Alpha 1-antitrypsin, COPD, Gene expression, Krüppel-like zinc finger proteins, Affymetrix gene chips

## Abstract

**Background:**

Chronic obstructive pulmonary disease (COPD) is influenced by environmental and genetic factors. An important fraction of COPD cases harbor a major genetic determinant, inherited ZZ (Glu342Lys) α1-antitrypsin deficiency (AATD). A study was undertaken to investigate gene expression patterns in end-stage COPD lungs from patients with and without AATD.

**Methods:**

Explanted lungs of end-stage ZZ AATD-related (treated and non-treated with AAT augmentation therapy) and “normal” MM COPD, and liver biopsies from patients suffering from liver cirrhosis with and without ZZ AATD were used for gene expression analysis by Affymetrix microarrays or RT-PCR.

**Results:**

A total of 162 genes were found to be differentially expressed (p-value ≤ 0.05 and |FC| ≥ 2) between MM and ZZ COPD patients. Of those, 134 gene sets were up-regulated and 28 were down-regulated in ZZ relative to MM lung tissue. A subgroup of genes, zinc finger protein 165, snail homolog 1 (Drosophila) (SNAI1), and Krüppel-like transcription factors (KLFs) 4 (gut), 9 and 10, perfectly segregated ZZ and MM COPD patients. The higher expression of KLF 9 and KLF10 has been verified in the replication cohort with AATD-related end-stage lung emphysema and liver cirrhosis. Furthermore, higher expression of KLF9, SNAI1 and DEFA1 was found in ZZ COPD lungs without augmentation therapy relative to MM COPD or ZZ COPD with augmentation therapy.

**Conclusions:**

These results reveal the involvement of transcriptional regulators of the zinc-finger family in COPD pathogenesis and provide deeper insight into the pathophysiological mechanisms of COPD with and without AATD.

## Background

Chronic obstructive pulmonary disease (COPD) includes a broad spectrum of respiratory symptoms and histopathological changes with varying degrees of severity, and it is typically diagnosed late in the course of disease when the patient presents with significant impairment [1,2]. Different inflammatory cells (macrophages, neutrophils, CD8+ T lymphocytes) and several interrelated processes, such as chronic inflammation per se, proteinase/anti-proteinase imbalance, oxidative stress, apoptosis and replenishment of structural cells, are thought to contribute to the pathogenesis of COPD [3]. Given this complexity of COPD, the identification of biological markers of disease susceptibility and/or progression is of great importance [4]. DNA microarrays have been proven to be a powerful tool capable of biomarker discovery for various disease entities. Previous studies used this approach to characterize global gene expression patterns from lung tissue of patients with COPD, and to identify genes associated with disease severity. So far, these gene expression patterns have produced differing results likely due to differences in the types of patients enrolled and due to inherent heterogeneity of COPD.

An important fraction of COPD cases harbor a major genetic determinant, inherited ZZ (Glu342Lys) α1-antitrypsin deficiency (AATD). AAT is an acute phase protein, main inhibitor of neutrophil elastase and a modulator of host inflammatory responses [5]. It is well established that the Z variant of AAT forms polymers and is retained in the endoplasmic reticulum (ER) of the hepatocytes [6] which leads to markedly impede (up to 90%) levels of the circulating AAT protein. The lack of AAT predisposes a person to develop early onset, rapidly progressive COPD where emphysema is a major component [7] whereas the accumulation of abnormally folded AAT protein increases the risk to develop chronic liver disease. Cirrhosis in ZZ AATD individuals may become clinically apparent at any age, with the peak incidence typically occurring in elderly never-smokers who have survived without developing severe emphysema [8].

In an attempt to further improve our understanding of the complex molecular basis of AATD-related pathology, we aimed to investigate gene expression patterns in well matched end-stage COPD lungs from patients with and without inherited α1-antitrypsin deficiency (AATD). The identified genes differently expressed were then validated in separate groups of ZZ and MM end-stage COPD lung tissue and biopsies from liver cirrhosis patients using real time PCR. This strategy was used to confirm the reproducibility of our findings in different populations, which increases the probability of results being applicable to the wider population of COPD patients.

## Methods

### Lung tissue

Tissues from explanted lungs of end-stage ZZ AATD-related COPD (n = 3) and non-AATD, MM COPD (n = 3) were received from the Biobank in Giessen which is a part of the EU 6th Framework Programme (Contract No: LSHM-CT-2005-018725, PULMOTENSION). This study has been approved by the local Ethics Committee (Marburg Ref. 198/7).

### RNA isolation and analysis by Affymetrix

Affymetrix analysis was performed at the University of Munich, Microarray Resource Centre, according to an established and approved protocol. Briefly, RNA was isolated from frozen lung tissues using the NucleoSpin RNA II kit from Macherey Nagel (Dueren, Germany), and quality aspects, such as absorbency ratios, 28 S/18 S ratios, and length of cDNA and cRNA synthesis products were assessed. Microarray hybridization probes were synthesized from 5 μg total RNA according to the manufacturer`s instructions (Affymetrix, Santa Clara, CA, USA). HumanHG-U133A Gene Chip arrays were hybridized and stained according to the protocol provided by Affymetrix. (http://www.mikrobio.med.tu-muenchen.de/category/expression-core-facility/).

The Affymetrix MAS 5.0 Suite 4.0 (MAS 4.0) software was used to calculate expression levels, fold change and to assess the significance of differential expression. A p-value < 0.05 was considered significant. Due to the exploratory nature of the microarray experiments, multiple testing correction was not performed [9]. Based on previous experiences, Affymetrix recommendations and published literature [10-13] transcripts exhibiting over 2-fold change were classed as being differentially expressed between ZZ and MM COPD samples. A further filtering step was introduced by assessing for significance of differential expression. The double filtered list [14] of differentially expressed genes (|FC| ≥ 2 and p-value ≤ 0.05) was further analysed using the Database for Annotation, Visualization and Integrated Discovery (DAVID) (http://david.abcc.ncifcrf.gov/). Based on the gene expression profiles from a specific functional annotation cluster, the pairwise Euclidean distance between the COPD profiles was calculated. The calculated distances were then visualized using multidimensional scaling (MDS) [15].

### Lung and liver tissue analysis by RT-PCR

The formalin-fixed and paraffin-embedded tissue samples (FFPE) were retrieved from the archives of the Institute of Pathology of Hannover Medical School following the requirements of the local Ethics Committee approval (Nr. 990–2011). We analysed surgical lung explants sampled at the time of transplantation from GOLD-4 MM COPD (n = 3) and ZZ COPD patients (n = 3), as well as liver biopsies from liver cirrhosis subjects with (n = 4) and without (n = 6) ZZ AATD. Selected tissue samples were (morphologically) matched to the above MM and ZZ COPD subgroups as closely as possible (Table 1A and B). In addition, we selected surgical lung explants from ZZ emphysema cases treated with AAT augmentation therapy (from 3 to 20 years; n = 11, age 52.5 ± 2.1 (SD) years) and non-treated with augmentation therapy (n = 6, age 44.7 ± 5.3 (SD) years). Lung explants from MM COPD cases (n = 6) served as a reference.

**Table 1:** Patient characteristics

**Variables**	**MM COPD**	**ZZ COPD**
	**N = 3**	**N = 3**
	*Mean ± SD*	
**A.** Transplanted lung tissue used for Affymetrix analysis		
Age (years)	57 ± 4.8	62 ± 2.3
Gender (male/female)	3/0	2/1
Ex-smoker	3	3
Gold stage	IV	IV
Mean pulmonary art. pressure (mmHg)	25 ±5.1	32 ±12
Cardiac Index ()	3.1 ± 0.3	3.0 ± 0.1
GOT (ref < 35U/L)	32 ± 21.1	28 ± 5.0
GPT (ref <45U/L)	25 ± 16.2	29 ± 8.0
Inhaled steroid therapy	3	3
Augmentation therapy with AAT	0	0
**B.** Transplanted lung tissue used for RT-PCR analysis		
Age (years)	53.7 ± 2.7	48.3 ± 1.3
Gender (male/female)	3/0	3/0
Ex-smoker	3	3
Gold stage	IV	IV
Mean pulmonary art. pressure (mmHg)	n.d.	28 ± 2.8
Cardiac Index ()	n.d.	n.d.
GOT (ref < 35U/L)	28 ±6.2	28.3 ± 3.4
GPT (ref <45U/L)	36.7 ± 10.2	33.3 ± 9.0
Inhaled steroid therapy	2	3
Augmentation therapy with AAT	0	0

### Laser-assisted microdissection and RNA extraction

FFPE 5 μm thick tissue sections were mounted on a poly-L-lysin-coated membrane attached to a special metal frame. After deparaffinization and hemalum staining, the CellCut Plus system (MMI Molecular Machines & Industries AG, Glattbrugg, Switzerland) was used for laser-assisted microdissection of lung compartments: we isolated sections of lung parenchyma that showed characteristic changes of end-stage emphysema using a no-touch technique, essentially as described [16].

### cDNA synthesis and pre-amplification

Complementary DNA of each sample was generated using the High Capacity cDNA Reverse Transcription Kit (Applied Biosystems, Foster City, CA, USA) and following the manufacturer’s protocol. Complementary DNA was pre-amplified in 14 PCR cycles with target gene-specific PCR primers, increasing the sensitivity of the subsequent real-time PCR analysis several thousand-fold (PreAmp Master Mix Kit, Applied Biosystems), as reported [17,18].

### Real-time PCR

The pre-amplified cDNA from microdissected specimens was evaluated by real-time PCR (TaqMan 7500 Real-Time PCR system, Applied Biosystems, Carlsbad, CA, USA). Quantification was performed in reactions containing pre-amplified cDNA, TaqMan Gene Expression Master Mix and the individual TaqMan Gene Expression Assay (both from Applied Biosystems). Target genes KLF9, KLF10, ZNF165, SNAIL1 and DEFA1 were selected and analysed, following the manufacturer’s protocol. For negative controls, the cDNA was replaced by water [19]. C_T_ values were calculated by normalization to the mean expression of three endogenous controls (POLR2A, β-GUS and GAPDH) and converted into 2^-ΔCT^ values with Excel 8.0 (Microsoft, Redmond, WA, USA) which were then statistically analysed using the Mann–Whitney *U* test and Prism 5.0. Values ≤ 0.05 were considered as statistically significant. Expression graphics were created using GraphPadPrism, version 5.0 (GraphPad Software, San Diego, CA, USA).

## Results

Tissues from explanted lungs of end-stage ZZ AATD-related COPD (n = 3) and non-AATD, MM COPD (n = 3) were used for gene expression analysis by Affymetrix. Detailed information about these patients can be found in Table 1 A. All patients had severe lung function impairment; however, none of the patients was a current smoker at the time of surgery and no one was treated with AAT augmentation therapy. Another cohort of lung specimens was used for laser-assisted microdissection, RNA extraction and RT-PCR analysis. Lung explants from MM COPD (n = 3) and ZZ COPD (n = 3) have been matched to the group of the cases used in Affymetrix analysis (Table 1 B). To avoid the regional variability of gene expression, we sampled lung tissue, several from central and peripheral regions. Liver biopsies were obtained from liver cirrhosis patients: 6 without [5 males and 1 female, mean age 56.8 ± 6.7 (SD)] and 4 with [males, mean age of 56.25 ± 6.9 (SD)] ZZ AATD. Additional RT-PCR analysis of selected genes was performed on surgical lung explants from end-stage ZZ emphysema cases with (n = 11, age 52.5 ± 2.1 (SD) years) and without AAT augmentation therapy (n = 6, age 44.7 ± 5.3 (SD) years).

### Genes that distinguish MM and ZZ end-stage COPD

A total of 162 probe sets were found to be differentially expressed (p-value ≤ 0.05 and |FC| ≥2) between MM “normal” and ZZ AATD-related end-stage COPD. Of those, 134 genes were up-regulated in ZZ lung tissue, while 28 were down-regulated (Additional file 1: Table S1). Notably, among the top 5 probe sets with highest expression values in ZZ versus MM lungs, we found defensin alpha1, nuclear receptor subfamily 4, hemoglobin gamma, and pentraxin-related gene. All these genes were previously associated with COPD [19-21].

### Gene ontology enrichment and functional group analysis

Using functional enrichment analysis according to the DAVID online resource [22], we identified functional groups of genes from tissue expression, diseases and biological processes (UP_TISSUE,GENETIC_ASSOCIATION_DB_DISEASE and GOTERM_BP_FAT) that are significantly more enriched (the most overrepresented) in ZZ versus MM COPD. As listed in Additional file 2: Table S2, our search for differential gene expression in end-stage COPD tissue from ZZ subjects revealed a significant overrepresentation of genes associated with specific tissue: 30 (22.9%) liver (p = 0.006), 11 (8.4%) skeletal muscle (p = 0.016), and 30 (22.9%) lung (p = 0.039). Analysis of disease-association, revealed a significantly enriched number of genes involved in diabetes type 2 (p = 0.013), Parkinson’s disease (p = 0.015), Alzheimer`s disease (p = 0.033) and polyneuropathy vasculitis (p = 0.033) (Additional file 3: Table S3). Furthermore, among all differentially expressed genes in ZZ versus MM COPD cases, 19 (14.5%, p < 0.001) genes were classified for the biological process of response to organic substance (GO:0010033), 20 (15.3%, p < 0.001) genes were classified for the biological process of regulation of cell proliferation (GO:0042127), 10 (7.6%, p < 0.001) genes were classified for the transmembrane receptor protein tyrosine kinase signalling pathway (GO:0007167), 15 (11.5%, p < 0.001) genes were classified for the biological process of response to injury (GO:0009611) and 12 (9.2%, p = 0.0013) genes were classified for the biological process of enzyme-linked receptor protein signalling pathway (GO:0007167) (Additional file 4: Table S4). The genes from the above-listed biological processes (GO) are presented in Table 2.

**Table 2:** GO Biological processes enriched in lung tissue of ZZ relative to MM COPD

**Term**	**Gene number (%)**	**P value**
GO:0010033 ~ response to organic substance	19 (14.5%)	<0.0001
GO:0042127 ~ regulation of cell proliferation	20 (15.3%)	<0.0001
GO:0007169 ~ transmembrane receptor protein tyrosine kinase signaling pathway	10 (7.6%)	<0.0001
GO:0009611 ~ response to wounding	15 (11.5%)	<0.0001
GO:0007167 ~ enzyme linked receptor protein signaling pathway	12 (9.2%)	0.0013
GO:0008285 ~ negative regulation of cell proliferation	12 (9.2%)	0.0015
GO:0008283 ~ cell proliferation	13 (9.9%)	0.0019
GO:0050878 ~ regulation of body fluid levels	7 (5.3%)	0.0028
GO:0050817 ~ coagulation	6 (4.6%)	0.0041

### Clustering of the genes

**Figure 1:**
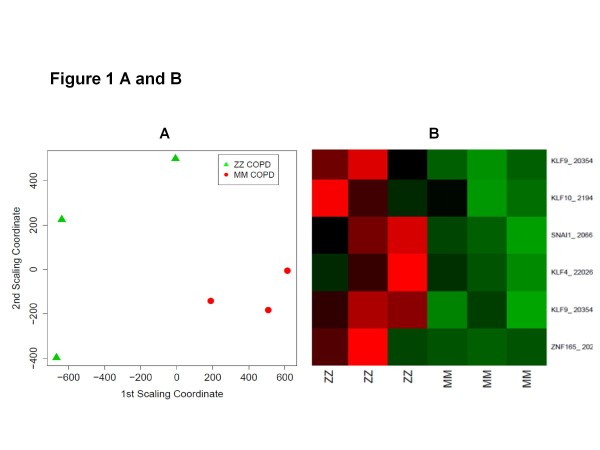
**A: A cluster of functionally related genes, as found by DAVID functional annotation classification, perfectly segregates MM and ZZ COPD samples. **This separation is visualized here by multidimensional scaling (MDS). **A**: Based on the gene expression patterns of the specific functional gene cluster, the pairwise Euclidean distance between the COPD samples is shown in a two-dimensional space. **B**: The visualization from Figure 1 A is further supported by a heatmap depiction. This heat map shows the relative (z-score) expression level for each gene and sample combination. A high expression level is associated with red, while green represents a low expression level.

**Figure 2:**
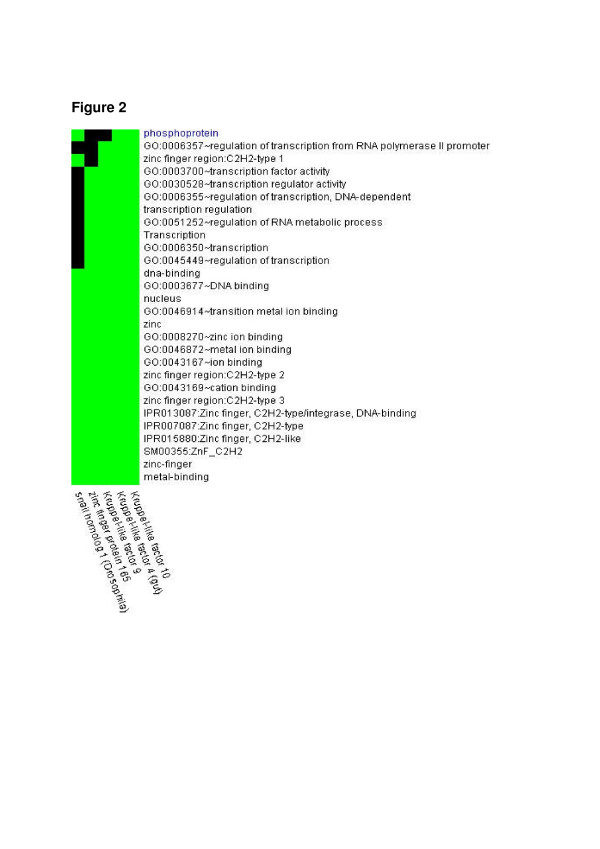
**One functionally related gene cluster allowed for the separation between MM and ZZ COPD samples (Figure**1**). **The figure shows the functional terms, based on which the genes of interest were grouped together. For each gene, membership of a functional group is color coded. Green signifies membership, while black states that the gene in question does not belong to the particular functional group.

Using functional annotation-based classification [22], we found 6 functionally related subgroups of genes. One subgroup perfectly segregated ZZ and MM COPD patients (Figure 1A and B). This set of genes included zinc finger protein 165, snail homolog 1 (Drosophila), Krüppel-like factors 4 (gut), 9 and 10 (Table 3), and DNA-binding transcriptional regulators that play diverse roles during differentiation and development. Functional similarity between the genes in the cluster is illustrated in Figure 2.

**Table 3:** Separating functional annotation group

**Affymetrix –ID**	**Gene name**	**Fold Change**	**p-value**
202393_s_at	Krüppel-like factor 10	−2,05	0,002
206683_at	zinc finger protein 165	−3,9	0,016
220266_s_at	Krüppel-like factor 4 (gut)	−2,3	0,036
219480_at	snail homolog 1 (Drosophila)	−2,08	0,001
203543_s_at, 203542_s_at	Krüppel-like factor 9	−3 (−2,77)	0,005 (0,02)

### Analysis of candidate genes by RT-PCR

**Figure 3:**
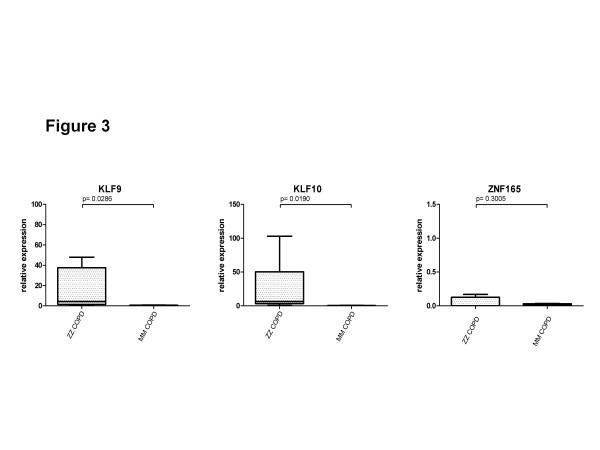
**mRNA expression of KLF9, KLF10 and ZNF165 in explanted lung tissue of end-stage ZZ AATD-related (n = 3) and “normal” MM COPD (n = 3). **Expression of target genes was calculated using the Δ C_T_-method (see Materials and Methods for details). Lines indicate median and percentiles, p-values indicate significance.

**Figure 4:**
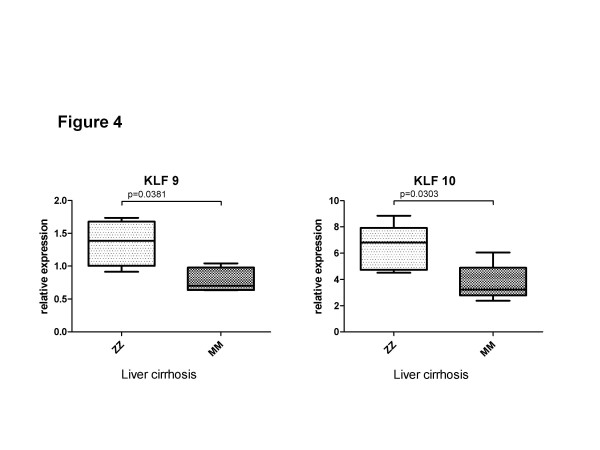
**mRNA expression of KLF9 and KLF10 in cirrhotic livers with (n = 4) and without (n = 6) ZZ AATD. **Expression of target genes was calculated using the Δ C_T_-method (see Materials and Methods for details). Lines indicate median and percentiles, p-values indicate significance.

Three candidate genes, KLF9, KLF10 and ZNF165, were randomly selected for validation by using a real-time PCR analysis in the 6 additional explanted COPD lungs from GOLD IV cases with and without ZZ AATD. Figure 3 verifies that the expression of KLF9 (33.7-fold, p = 0.0286) and KLF10 (98.5-fold, p = 0.019) is significantly higher in end-stage ZZ compared to MM COPD lungs. The expression of ZNF165 was very low in all analysed lung tissues but was higher by 50% (NS) in ZZ than in MM cases. To further validate whether differential expression of these genes is linked to the ZZ AATD, we analysed liver tissues from cirrhosis patients with and without ZZ AATD. Both KLF9 (1.7-fold, p = 0.038) and KLF10 (1.73-fold, p = 0.030) were found to be significantly higher expressed in liver cirrhosis tissue from ZZ compared to MM AAT (Figure 4). The relative expression levels of ZNF165 in the analysed samples were very low or below detection limits (data not shown).

**Figure 5:**
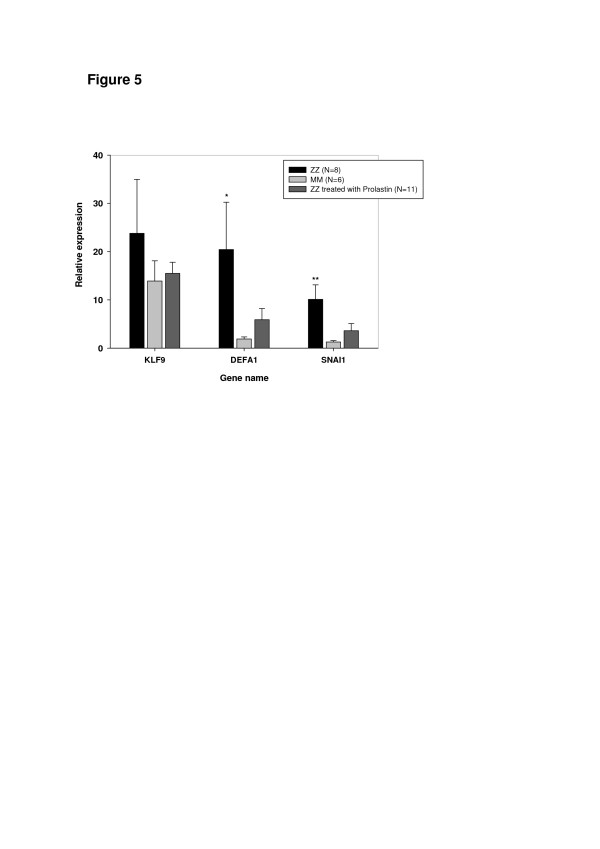
**Relative mRNA levels for selected genes in lung explants from ZZ AAT deficiency-related emphysema patients treated from 3 to 20 years with AAT augmentation therapy compared to samples from matched, but non-treated patients and MM COPD controls. **See Methods for details on microdissection, RNA extraction and RT-PCR. Bars indicate the mean (SE), *p < 0.05 and **p < 0.01.

To further confirm our findings, lung tissue samples obtained from ZZ COPD with and without augmentation therapy, were analysed for the expression of KLF9, SNAIL1 and DEFA1. Remarkably, surgical lung explants obtained from ZZ AAT deficiency-related emphysema patients treated from 3 to 20 years with AAT augmentation therapy showed lower expression of these genes – and did not differ significantly from MM specimens (Figure 5). Moreover, DEFA1 and SNAIL1 were found to be significantly higher expressed in ZZ end-stage lungs as compared to MM (n = 6; 10.98-fold, p = 0.05 and 7.87-fold, p = 0.01, respectively). KLF9 was also higher expressed in ZZ (by 1.5-fold, NS) relative to MM COPD, although these differences did not reached statistical significance.

## Discussion

To further explore lung tissue analysis using microarrays, we examined gene expression profiles in well matched end-stage (GOLD IV) COPD cases with ZZ AATD and without, “normal” MM COPD. Primarily, we wanted to find genes differentially expressed between MM and ZZ end-stage COPD and secondly, we sought for probable cluster(s) of genes which are able to stratify COPD cases by the genetic variant of AAT. It is important to point out that most patients with AATD-related emphysema are treated with AAT augmentation therapy. In order to make an appropriate match with “normal” MM COPD, we included non-augmented AATD cases which very much restricted the sample size of our cohort. We therefore deliberately used different cohorts of COPD and liver cirrhosis patients for the RT-PCR, because the replication of gene expression findings in a distinct cohort increases the likelihood of these results being applicable to wider populations of COPD patients.

Nevertheless, our results show that a relatively small number of genes is significantly differently expressed in end-stage ZZ lungs when compared to those from MM. Remarkably, out of 162 gene sets differently expressed between ZZ and MM-COPD lungs, 134 (82.7%) were up-regulated and only 28 (17.3%) were down-regulated in ZZ lungs. It is worth pointing out that among all up-regulated gene sets, the gene encoding α1-defensin (DEFA1) was one of the most highly (13-fold higher) expressed in ZZ compared to MM lung tissue. This finding was confirmed by RT-PCR analysis and is in line with previous studies showing that α-defensin concentrations are increased in individuals with AATD [23] and that patients with AATD have a milieu where high concentrations of α-defensins and low amounts of AAT potentiate the impact of α-defensins on lung injury [24,25]. Remarkably, we could further verify in different cohorts that the expression of DEFA1 is higher in lungs from ZZ cases compared to MM COPD specimens. Based on this latter finding, we examined whether ZZ AAT deficiency-related emphysema patients treated with Prolastin® had lower DEFA1 expression in their lungs. Indeed, we found that augmentation therapy lowers DEFA1 expression when compared to untreated matched patients. Thus, our discovery that AAT influences DEFA1 gene expression opens new possibilities to further optimize augmentation therapies.

To our surprise, a set of genes including zinc finger protein 165, snail homolog 1 (Drosophila), and Krüppel-like transcription factors (KLFs) 4 (gut), 9 and 10, perfectly discriminated between patients with end-stage “normal” MM and ZZ AATD–related COPD (Figures 1 and 2). Further conformation in a different cohort of end-stage COPD lungs and in liver cirrhosis tissues showing that SNAI1, KLF9 and KLF10 are higher expressed in ZZ relative to MM AAT subjects, allows us to propose that the increased expression of zinc finger transcription factors is related to AATD. To our knowledge, so far only one study has shown an enhanced expression (2.1-fold) of Krüppel-related zinc finger protein (specifically undefined) in lung tissue from patients with AATD-associated emphysema when compared to normal lung tissue [26] and it has been shown that KLF4 mRNA is up-regulated more than 10-fold in mice lung tissue after endotoxin stimuli [27]. KLF10 was initially identified as a primary transforming growth factor beta (TGF-β)-inducible early gene in human osteoblasts [23]. KLF10 gene expression in the liver is shown to be regulated by glucose. This argues for a pleiotropic role of KLF10 in cellular biology by integrating antiproliferative, endocrine, metabolic, and circadian signals. As a matter of fact, genomic analysis of the liver in a transgenic mouse model with inducible expression of mutant Z and normal M variant of AAT protein unveiled a number of significantly more highly expressed genes in Z mice as an indicative of a TGF-β effect (among others KLF10) [28]. These data, together with our current findings suggest that induction of KLF10 gene expression might be specific for the ER stress state that is associated with the intracellular accumulation of aggregated Z AAT protein in the ER.

KLFs are critical regulators of many physiological processes and are involved in disorders such as cardiovascular disease, cancer, and inflammatory conditions. While numerous zinc finger proteins are expressed in the lung, only a few have been clearly implicated in lung homeostasis and disease. A recent study by Bhattacharya and co-workers [29] identified biomarker genes enriched in functions related to DNA binding and regulation of transcription which allow the prediction of disease in an unrelated data set, generated from patients with severe emphysema, with 97% accuracy. Although individual biomarkers in this study have not been identified, it is clearly shown that the selected genes include members of zinc finger–binding domain containing proteins. The patterns of KLF gene expression have not been considered in the pathobiology of AATD-associated COPD and warrants further investigation.

## Conclusions

Lungs with end-stage emphysema display decreases in overall global gene expression, with pronounced increase in transcripts encoding pro-inflammatory mediators, immune-response proteins, and proteolytic enzymes. Some of these differentially-expressed genes are specifically linked to ZZ AATD-related emphysema. Results from the current study revealed a subgroup of genes from the zinc-finger family which perfectly segregated ZZ and MM COPD patients. This finding not only shows that these two types of emphysema can be distinguished from each other, but also provides a deeper insight into the pathophysiological mechanisms of lung emphysema with and without AATD.

## Competing interests

The authors have no conflicts of interest to disclose.

A.-Rembert Koczulla: Has received speaker fees and travel support from Grifols (former Talecris Biotherapeutics).

Sarah Noeske: Has received travel support from Grifols.

Christian Herr: Has received travel support from Grifols.

Claus Vogelmeier: Has received speaker fees, research grants and consultancy fees from Astra Zeneca, Boehringer, Chiesi, Glaxo Smith Kline, Janssen-Cilag, Mundipharma, Novartis, Nycomed, Pfizer, Grifols.

Sabina Janciauskiene: Has received speaker fees, research grants and consultancy fees from Grifols.

Thomas Wolf has no conflicts of interest to disclose.

Nils von Neuhoff has no conflicts of interest to disclose.

Walter Klepetko has no conflicts of interest to disclose.

Heiko Golpon, has no conflicts of interest to disclose.

Robert Vosswinkel has no conflicts of interest to disclose.

Maurizio Luisetti speaker fees,research grants and consultancy and travel support from Grifols, Kedrion SpA, Nycomed, GSK, and Boehringer Ingelheim.

Ilaria Ferrarotti speaker and consultancy fees and travel support from Grifols and Kedrion SpA.

Tobias Welte speaker fees and travel support from Grifols.

Johanna Rische has no conflicts of interest to disclose.

Sabine Wrenger has no conflicts of interest to disclose.

Danny Jonigk has no conflicts of interest to disclose.

## Authors’ contributions

RK, DJ, SJ, ThW, ChH, SN, WK, JR-have made substantial contributions to conception and design, or acquisition of data, or analysis and interpretation of data; CV, NN, SW, HG, RV, ML, IF, TW- have been involved in drafting the manuscript or revising it critically for important intellectual content. All authors read and approved the final manuscript.

## Supplementary Material

Additional file 1 **Table S1. **Total of 162 probe sets differentially expressed (p-value ≤ 0.05 and |FC| ≥2) between MM “normal” and ZZ AATD-related end-stage COPD. Click here for file

Additional file 2 **Table S2. **Tissue and cell-associated genes enriched in end-stage COPD lung tissue from ZZ compared to MM AAT patients. Click here for file

Additional file 3 **Table S3. **Disease-associated genes enriched in end-stage COPD lung tissue from ZZ compared to MM AAT patients. Click here for file

Additional file 4 **Table S4. **More highly expressed genes associated with biological processes in end-stage COPD lung tissue from ZZ compared to MM AAT patients. Click here for file
