# Supplementary material for: Krüppel-like zinc finger proteins in end-stage COPD lungs with and without severe alpha1-antitrypsin deficiency
Source: Orphanet J Rare Dis. 2012 May 23;7:29. doi: 10.1186/1750-1172-7-29 (PMC3517304; doi:10.1186/1750-1172-7-29)
Supplement: Additional file 1 — Table S1. Total of 162 probe sets differentially expressed (p-value ≤ 0.05 and |FC| ≥2) between MM “normal” and ZZ AATD-related end-stage COPD. [file 1750-1172-7-29-S1.doc]

**Supplement Table 1**. Total of 162 probe sets differentially expressed (p-value ≤ 0.05 and |FC| ≥2) between MM “normal” and ZZ AATD-related end-stage COPD.

| **N** | **Probe set** | **FC MM vs ZZ**  **COPD** | **Gene** | **Gene Symbol** |
| --- | --- | --- | --- | --- |
| 1 | 205033_s_at | -13,23 | defensin, alpha 1 /// defensin, alpha 3, neutrophil-specific /// similar to Neutrophil defensin 1 precursor (HNP-1) (HP-1) (HP1) (Defensin, alpha 1) | DEFA1 /// DEFA3 /// LOC653600 |
| 2 | 209959_at | -10,36 | nuclear receptor subfamily 4, group A, member 3 | NR4A3 |
| 3 | 207978_s_at | -10,33 | nuclear receptor subfamily 4, group A, member 3 | NR4A3 |
| 4 | 204419_x_at | -9,98 | hemoglobin, gamma A /// hemoglobin, gamma A /// hemoglobin, gamma G /// hemoglobin, gamma G | HBG1 /// HBG2 |
| 5 | 206157_at | -8,48 | pentraxin-related gene, rapidly induced by IL-1 beta | PTX3 |
| 6 | 220287_at | -8,24 | ADAM metallopeptidase with thrombospondin type 1 motif, 9 | ADAMTS9 |
| 7 | 205239_at | -7,26 | amphiregulin (schwannoma-derived growth factor) /// similar to Amphiregulin precursor (AR) (Colorectum cell-derived growth factor) (CRDGF) | AREG /// LOC653193 |
| 8 | 205767_at | -6,82 | epiregulin | EREG |
| 9 | 213895_at | -6,65 | epithelial membrane protein 1 | EMP1 |
| 10 | 221031_s_at | -5,70 | apolipoprotein L domain containing 1 /// apolipoprotein L domain containing 1 | APOLD1 |
| 11 | 213076_at | -5,32 | inositol 1,4,5-trisphosphate 3-kinase C | ITPKC |
| 12 | 217546_at | -5,19 | metallothionein 1M | MT1M |
| 13 | 201109_s_at | -4,78 | thrombospondin 1 | THBS1 |
| 14 | 204575_s_at | -4,72 | matrix metallopeptidase 19 /// similar to Matrix metalloproteinase-19 precursor (MMP-19) (Matrix metalloproteinase RASI) (MMP-18) | MMP19 /// LOC652543 |
| 15 | 201325_s_at | -4,68 | epithelial membrane protein 1 | EMP1 |
| 16 | 218880_at | -4,57 | FOS-like antigen 2 | FOSL2 |
| 17 | 217739_s_at | -4,52 | pre-B-cell colony enhancing factor 1 /// similar to Nicotinamide phosphoribosyltransferase (NAmPRTase) (Nampt) (Pre-B cell enhancing factor) (Pre-B-cell colony-enhancing factor 1) /// pre-B cell enhancing factor 1 pseudogene | PBEF1 /// LOC646309 /// RP11-92J19,4 |
| 18 | 205193_at | -4,17 | v-maf musculoaponeurotic fibrosarcoma oncogene homolog F (avian) | MAFF |
| 19 | 203372_s_at | -4,03 | suppressor of cytokine signaling 2 | SOCS2 |
| 20 | 201108_s_at | -3,95 | thrombospondin 1 | THBS1 |
| 21 | 206683_at | -3,90 | zinc finger protein 165 | ZNF165 |
| 22 | 219993_at | -3,89 | SRY (sex determining region Y)-box 17 | SOX17 |
| 23 | 36711_at | -3,85 | v-maf musculoaponeurotic fibrosarcoma oncogene homolog F (avian) | MAFF |
| 24 | 201110_s_at | -3,80 | thrombospondin 1 | THBS1 |
| 25 | 203574_at | -3,66 | nuclear factor, interleukin 3 regulated | NFIL3 |
| 26 | 206390_x_at | -3,65 | platelet factor 4 (chemokine (C-X-C motif) ligand 4) | PF4 |
| 27 | 209681_at | -3,65 | solute carrier family 19 (thiamine transporter), member 2 | SLC19A2 |
| 28 | 210773_s_at | -3,62 | formyl peptide receptor-like 1 /// formyl peptide receptor-like 1 | FPRL1 |
| 29 | 205756_s_at | -3,46 | coagulation factor VIII, procoagulant component (hemophilia A) | F8 |
| 30 | 204607_at | -3,46 | 3-hydroxy-3-methylglutaryl-Coenzyme A synthase 2 (mitochondrial) | HMGCS2 |
| 31 | 203438_at | -3,45 | stanniocalcin 2 | STC2 |
| 32 | 220187_at | -3,42 | STEAP family member 4 | STEAP4 |
| 33 | 222162_s_at | -3,34 | ADAM metallopeptidase with thrombospondin type 1 motif, 1 | ADAMTS1 |
| 34 | 217738_at | -3,31 | pre-B-cell colony enhancing factor 1 /// similar to Nicotinamide phosphoribosyltransferase (NAmPRTase) (Nampt) (Pre-B cell enhancing factor) (Pre-B-cell colony-enhancing factor 1) /// pre-B cell enhancing factor 1 pseudogene | PBEF1 /// LOC646309 /// RP11-92J19,4 |
| 35 | 219911_s_at | -3,28 | solute carrier organic anion transporter family, member 4A1 | SLCO4A1 |
| 36 | 210772_at | -3,18 | formyl peptide receptor-like 1 /// formyl peptide receptor-like 1 | FPRL1 |
| 37 | 205863_at | -3,13 | S100 calcium binding protein A12 (calgranulin C) /// S100 calcium binding protein A12 (calgranulin C) | S100A12 |
| 38 | 201324_at | -3,12 | epithelial membrane protein 1 | EMP1 |
| 39 | 218319_at | -3,04 | pellino homolog 1 (Drosophila) | PELI1 |
| 40 | 210873_x_at | -3,03 | apolipoprotein B mRNA editing enzyme, catalytic polypeptide-like 3A | APOBEC3A |
| 41 | 203373_at | -3,01 | suppressor of cytokine signaling 2 | SOCS2 |
| 42 | 203543_s_at | -3,00 | Kruppel-like factor 9 | KLF9 |
| 43 | 203946_s_at | -2,97 | arginase, type II | ARG2 |
| 44 | 203499_at | -2,94 | EPH receptor A2 | EPHA2 |
| 45 | 823_at | -2,91 | chemokine (C-X3-C motif) ligand 1 | CX3CL1 |
| 46 | 207630_s_at | -2,89 | cAMP responsive element modulator | CREM |
| 47 | 203687_at | -2,84 | chemokine (C-X3-C motif) ligand 1 | CX3CL1 |
| 48 | 212665_at | -2,84 | TCDD-inducible poly(ADP-ribose) polymerase | TIPARP |
| 49 | 210517_s_at | -2,84 | A kinase (PRKA) anchor protein (gravin) 12 | AKAP12 |
| 50 | 209967_s_at | -2,81 | cAMP responsive element modulator | CREM |
| 51 | 220001_at | -2,79 | peptidyl arginine deiminase, type IV | PADI4 |
| 52 | 221796_at | -2,79 | neurotrophic tyrosine kinase, receptor, type 2 | NTRK2 |
| 53 | 203542_s_at | -2,77 | Kruppel-like factor 9 | KLF9 |
| 54 | 210827_s_at | -2,76 | E74-like factor 3 (ets domain transcription factor, epithelial-specific ) | ELF3 |
| 55 | 215047_at | -2,75 | tripartite motif-containing 58 | TRIM58 |
| 56 | 202067_s_at | -2,70 | low density lipoprotein receptor (familial hypercholesterolemia) | LDLR |
| 57 | 217996_at | -2,69 | pleckstrin homology-like domain, family A, member 1 | PHLDA1 |
| 58 | 202376_at | -2,66 | serpin peptidase inhibitor, clade A (alpha-1 antiproteinase, antitrypsin), member 3 | SERPINA3 |
| 59 | 220370_s_at | -2,65 | ubiquitin specific peptidase 36 | USP36 |
| 60 | 203835_at | -2,65 | leucine rich repeat containing 32 | LRRC32 |
| 61 | 204784_s_at | -2,61 | myeloid leukemia factor 1 | MLF1 |
| 62 | 222164_at | -2,61 | fibroblast growth factor receptor 1 (fms-related tyrosine kinase 2, Pfeiffer syndrome) | FGFR1 |
| 63 | 217997_at | -2,61 | pleckstrin homology-like domain, family A, member 1 | PHLDA1 |
| 64 | 204846_at | -2,58 | ceruloplasmin (ferroxidase) | CP |
| 65 | 217173_s_at | -2,58 | low density lipoprotein receptor (familial hypercholesterolemia) | LDLR |
| 66 | 214508_x_at | -2,55 | cAMP responsive element modulator | CREM |
| 67 | 202068_s_at | -2,54 | low density lipoprotein receptor (familial hypercholesterolemia) | LDLR |
| 68 | 209183_s_at | -2,54 | chromosome 10 open reading frame 10 | C10orf10 |
| 69 | 214918_at | -2,52 | heterogeneous nuclear ribonucleoprotein M | HNRPM |
| 70 | 217911_s_at | -2,52 | BCL2-associated athanogene 3 | BAG3 |
| 71 | 203737_s_at | -2,51 | peroxisome proliferative activated receptor, gamma, coactivator-related 1 | PPRC1 |
| 72 | 209182_s_at | -2,51 | chromosome 10 open reading frame 10 | C10orf10 |
| 73 | 202643_s_at | -2,49 | tumor necrosis factor, alpha-induced protein 3 | TNFAIP3 |
| 74 | 202628_s_at | -2,47 | serpin peptidase inhibitor, clade E (nexin, plasminogen activator inhibitor type 1), member 1 | SERPINE1 |
| 75 | 221489_s_at | -2,47 | sprouty homolog 4 (Drosophila) /// similar to sprouty homolog 4 (Drosophila) | SPRY4 /// LOC653170 |
| 76 | 207674_at | -2,46 | Fc fragment of IgA, receptor for | FCAR |
| 77 | 209758_s_at | -2,45 | microfibrillar associated protein 5 | MFAP5 |
| 78 | 204363_at | -2,45 | coagulation factor III (thromboplastin, tissue factor) | F3 |
| 79 | 205027_s_at | -2,45 | mitogen-activated protein kinase kinase kinase 8 | MAP3K8 |
| 80 | 218881_s_at | -2,45 | FOS-like antigen 2 | FOSL2 |
| 81 | 215350_at | -2,43 | spectrin repeat containing, nuclear envelope 1 | SYNE1 |
| 82 | 209555_s_at | -2,43 | CD36 molecule (thrombospondin receptor) | CD36 |
| 83 | 215990_s_at | -2,42 | B-cell CLL/lymphoma 6 (zinc finger protein 51) | BCL6 |
| 84 | 205409_at | -2,41 | FOS-like antigen 2 | FOSL2 |
| 85 | 205214_at | -2,41 | serine/threonine kinase 17b (apoptosis-inducing) | STK17B |
| 86 | 202238_s_at | -2,40 | nicotinamide N-methyltransferase | NNMT |
| 87 | 202147_s_at | -2,39 | interferon-related developmental regulator 1 | IFRD1 |
| 88 | 206036_s_at | -2,38 | v-rel reticuloendotheliosis viral oncogene homolog (avian) | REL |
| 89 | 207822_at | -2,33 | fibroblast growth factor receptor 1 (fms-related tyrosine kinase 2, Pfeiffer syndrome) | FGFR1 |
| 90 | 202150_s_at | -2,33 | neural precursor cell expressed, developmentally down-regulated 9 | NEDD9 |
| 91 | 220266_s_at | -2,32 | Kruppel-like factor 4 (gut) | KLF4 |
| 92 | 204715_at | -2,32 | pannexin 1 | PANX1 |
| 93 | 214446_at | -2,29 | elongation factor, RNA polymerase II, 2 | ELL2 |
| 94 | 219937_at | -2,29 | thyrotropin-releasing hormone degrading enzyme | TRHDE |
| 95 | 200796_s_at | -2,28 | myeloid cell leukemia sequence 1 (BCL2-related) | MCL1 |
| 96 | 206515_at | -2,26 | cytochrome P450, family 4, subfamily F, polypeptide 3 | CYP4F3 |
| 97 | 200798_x_at | -2,24 | myeloid cell leukemia sequence 1 (BCL2-related) | MCL1 |
| 98 | 207030_s_at | -2,24 | cysteine and glycine-rich protein 2 | CSRP2 |
| 99 | 207057_at | -2,23 | solute carrier family 16 (monocarboxylic acid transporters), member 7 | SLC16A7 |
| 100 | 214056_at | -2,23 | Myeloid cell leukemia sequence 1 (BCL2-related) | MCL1 |
| 101 | 201329_s_at | -2,20 | v-ets erythroblastosis virus E26 oncogene homolog 2 (avian) | ETS2 |
| 102 | 220330_s_at | -2,19 | SAM domain, SH3 domain and nuclear localisation signals, 1 | SAMSN1 |
| 103 | 208078_s_at | -2,19 | SNF1-like kinase /// SNF1-like kinase | SNF1LK |
| 104 | 212614_at | -2,19 | AT rich interactive domain 5B (MRF1-like) | ARID5B |
| 105 | 203888_at | -2,17 | thrombomodulin | THBD |
| 106 | 202464_s_at | -2,16 | 6-phosphofructo-2-kinase/fructose-2,6-biphosphatase 3 | PFKFB3 |
| 107 | 220576_at | -2,15 | GPI deacylase | PGAP1 |
| 108 | 211538_s_at | -2,15 | heat shock 70kDa protein 2 | HSPA2 |
| 109 | 202644_s_at | -2,14 | tumor necrosis factor, alpha-induced protein 3 | TNFAIP3 |
| 110 | 204769_s_at | -2,13 | transporter 2, ATP-binding cassette, sub-family B (MDR/TAP) | TAP2 |
| 111 | 205932_s_at | -2,13 | msh homeobox homolog 1 (Drosophila) | MSX1 |
| 112 | 204258_at | -2,12 | chromodomain helicase DNA binding protein 1 | CHD1 |
| 113 | 204224_s_at | -2,12 | GTP cyclohydrolase 1 (dopa-responsive dystonia) | GCH1 |
| 114 | 207547_s_at | -2,12 | family with sequence similarity 107, member A | FAM107A |
| 115 | 203939_at | -2,11 | 5'-nucleotidase, ecto (CD73) | NT5E |
| 116 | 205729_at | -2,11 | oncostatin M receptor | OSMR |
| 117 | 210287_s_at | -2,10 | fms-related tyrosine kinase 1 (vascular endothelial growth factor/vascular permeability factor receptor) | FLT1 |
| 118 | 201161_s_at | -2,10 | cold shock domain protein A | CSDA |
| 119 | 219480_at | -2,08 | snail homolog 1 (Drosophila) | SNAI1 |
| 120 | 221704_s_at | -2,05 | vacuolar protein sorting 37 homolog B (S, cerevisiae) /// vacuolar protein sorting 37 homolog B (S, cerevisiae) | VPS37B |
| 121 | 219140_s_at | -2,05 | retinol binding protein 4, plasma | RBP4 |
| 122 | 202393_s_at | -2,05 | Kruppel-like factor 10 | KLF10 |
| 123 | 221477_s_at | -2,04 | hypothetical protein MGC5618 | MGC5618 |
| 124 | 211580_s_at | -2,04 | phosphoinositide-3-kinase, regulatory subunit 3 (p55, gamma) | PIK3R3 |
| 125 | 202146_at | -2,03 | interferon-related developmental regulator 1 | IFRD1 |
| 126 | 213006_at | -2,03 | CCAAT/enhancer binding protein (C/EBP), delta | CEBPD |
| 127 | 219106_s_at | -2,03 | kelch repeat and BTB (POZ) domain containing 10 | KBTBD10 |
| 128 | 205695_at | -2,02 | serine dehydratase | SDS |
| 129 | 203140_at | -2,02 | B-cell CLL/lymphoma 6 (zinc finger protein 51) /// B-cell CLL/lymphoma 6 (zinc finger protein 51) | BCL6 |
| 130 | 204439_at | -2,02 | interferon-induced protein 44-like | IFI44L |
| 131 | 211126_s_at | -2,02 | cysteine and glycine-rich protein 2 | CSRP2 |
| 132 | 210090_at | -2,02 | activity-regulated cytoskeleton-associated protein | ARC |
| 133 | 203887_s_at | -2,02 | thrombomodulin | THBD |
| 134 | 219250_s_at | -2,01 | fibronectin leucine rich transmembrane protein 3 | FLRT3 |
| 135 | 202437_s_at | 2,03 | cytochrome P450, family 1, subfamily B, polypeptide 1 | CYP1B1 |
| 136 | 219344_at | 2,07 | solute carrier family 29 (nucleoside transporters), member 3 | SLC29A3 |
| 137 | 204684_at | 2,09 | neuronal pentraxin I | NPTX1 |
| 138 | 209686_at | 2,09 | S100 calcium binding protein, beta (neural) | S100B |
| 139 | 204689_at | 2,09 | homeobox, hematopoietically expressed | HHEX |
| 140 | 204039_at | 2,10 | CCAAT/enhancer binding protein (C/EBP), alpha | CEBPA |
| 141 | 220625_s_at | 2,12 | E74-like factor 5 (ets domain transcription factor) | ELF5 |
| 142 | 209571_at | 2,14 | CBF1 interacting corepressor | CIR |
| 143 | 205738_s_at | 2,18 | fatty acid binding protein 3, muscle and heart (mammary-derived growth inhibitor) | FABP3 |
| 144 | 215784_at | 2,18 | CD1e molecule | CD1E |
| 145 | 210549_s_at | 2,22 | chemokine (C-C motif) ligand 23 | CCL23 |
| 146 | 221584_s_at | 2,28 | potassium large conductance calcium-activated channel, subfamily M, alpha member 1 | KCNMA1 |
| 147 | 202436_s_at | 2,31 | cytochrome P450, family 1, subfamily B, polypeptide 1 | CYP1B1 |
| 148 | 206420_at | 2,32 | immunoglobulin superfamily, member 6 | IGSF6 |
| 149 | 215933_s_at | 2,35 | homeobox, hematopoietically expressed | HHEX |
| 150 | 215867_x_at | 2,40 | carbonic anhydrase XII | CA12 |
| 151 | 201387_s_at | 2,47 | ubiquitin carboxyl-terminal esterase L1 (ubiquitin thiolesterase) | UCHL1 |
| 152 | 215169_at | 2,56 | solute carrier family 35, member E2 | SLC35E2 |
| 153 | 214164_x_at | 2,73 | carbonic anhydrase XII | CA12 |
| 154 | 221266_s_at | 2,74 | transmembrane 7 superfamily member 4 /// transmembrane 7 superfamily member 4 | TM7SF4 |
| 155 | 219837_s_at | 2,85 | cytokine-like 1 | CYTL1 |
| 156 | 209987_s_at | 2,88 | achaete-scute complex-like 1 (Drosophila) | ASCL1 |
| 157 | 203963_at | 2,99 | carbonic anhydrase XII | CA12 |
| 158 | 205048_s_at | 3,14 | phosphoserine phosphatase | PSPH |
| 159 | 204284_at | 3,27 | protein phosphatase 1, regulatory (inhibitor) subunit 3C | PPP1R3C |
| 160 | 210809_s_at | 3,28 | periostin, osteoblast specific factor | POSTN |
| 161 | 210325_at | 3,92 | CD1a molecule | CD1A |
| 162 | 220380_at | 4,35 | deoxyribonuclease II beta | DNASE2B |

N, number; FC, fold change; COPD, chronic obstructive pulmonary disease. *Yellow marked* rows show genes that are higher expressed in MM COPD versus ZZ COPD.
