# Supplementary material for: Krüppel-like zinc finger proteins in end-stage COPD lungs with and without severe alpha1-antitrypsin deficiency
Source: Orphanet J Rare Dis. 2012 May 23;7:29. doi: 10.1186/1750-1172-7-29 (PMC3517304; doi:10.1186/1750-1172-7-29)
Supplement: Additional file 2 — Table S2. Tissue and cell-associated genes enriched in end-stage COPD lung tissue from ZZ compared to MM AAT patients. [file 1750-1172-7-29-S2.doc]

**Supplement Table 2**. Tissue and cell-associated genes enriched in end-stage COPD lung tissue from ZZ compared to MM AAT patients.

| **Term** | **Gene name** | **Gene Symbol** | **Gene number (%)** | **P value** |
| --- | --- | --- | --- | --- |
| **Liver 30 (22.9%) 0.006** | | | | |
|  | microfibrillar associated protein 5 | [MFAP5](http://www.genecards.org/cgi-bin/carddisp.pl?gene=MFAP5) |  |  |
|  | retinol binding protein 4, plasma | [RBP4](http://www.genecards.org/cgi-bin/carddisp.pl?gene=RBP4) |  |  |
|  | periostin, osteoblast specific factor | [POSTN](http://www.genecards.org/cgi-bin/carddisp.pl?gene=POSTN) |  |  |
|  | STEAP family member 4 | [STEAP4](http://www.genecards.org/cgi-bin/carddisp.pl?gene=STEAP4) |  |  |
|  | fatty acid binding protein 3, muscle and heart (mammary-derived growth inhibitor) | [FABP3](http://www.genecards.org/cgi-bin/carddisp.pl?gene=FABP3) |  |  |
|  | serine dehydratase | [SDS](http://www.genecards.org/cgi-bin/carddisp.pl?gene=SDS) |  |  |
|  | defensin, alpha 1 | [DEFA1](http://www.genecards.org/cgi-bin/carddisp.pl?gene=DEFA1) |  |  |
|  | nuclear receptor subfamily 4, group A, member 3 | [NR4A3](http://www.genecards.org/cgi-bin/carddisp.pl?gene=NR4A3) |  |  |
|  | nuclear receptor subfamily 4, group A, member 3 | [NR4A3](http://www.genecards.org/cgi-bin/carddisp.pl?gene=NR4A3) |  |  |
|  | CD36 molecule (thrombospondin receptor) | [CD36](http://www.genecards.org/cgi-bin/carddisp.pl?gene=CD36) |  |  |
|  | 5'-nucleotidase, ecto (CD73) | [NT5E](http://www.genecards.org/cgi-bin/carddisp.pl?gene=NT5E) |  |  |
|  | fibroblast growth factor receptor 1 | [RPS20P22](http://www.genecards.org/cgi-bin/carddisp.pl?gene=RPS20P22) |  |  |
|  | fibroblast growth factor receptor 1 | [FGFR1](http://www.genecards.org/cgi-bin/carddisp.pl?gene=FGFR1) |  |  |
|  | thrombospondin 1 | [THBS1](http://www.genecards.org/cgi-bin/carddisp.pl?gene=THBS1) |  |  |
|  | thrombospondin 1 | [THBS1](http://www.genecards.org/cgi-bin/carddisp.pl?gene=THBS1) |  |  |
|  | thrombospondin 1 | [THBS1](http://www.genecards.org/cgi-bin/carddisp.pl?gene=THBS1) |  |  |
|  | 3-hydroxy-3-methylglutaryl-coenzyme A synthase 2 (mitochondrial) | [HMGCS2](http://www.genecards.org/cgi-bin/carddisp.pl?gene=HMGCS2) |  |  |
|  | chemokine (C-C motif) ligand 23 | [CCL23](http://www.genecards.org/cgi-bin/carddisp.pl?gene=CCL23) |  |  |
|  | serpin peptidase inhibitor, clade A (alpha-1 antiproteinase, antitrypsin), member 3 | [SERPINA3](http://www.genecards.org/cgi-bin/carddisp.pl?gene=SERPINA3) |  |  |
|  | epithelial membrane protein 1 | [EMP1](http://www.genecards.org/cgi-bin/carddisp.pl?gene=EMP1) |  |  |
|  | epithelial membrane protein 1 | [Hs.436298](http://www.ncbi.nlm.nih.gov/UniGene/clust.cgi?ORG=Hs&CID=436298) |  |  |
|  | epithelial membrane protein 1 | [EMP1](http://www.genecards.org/cgi-bin/carddisp.pl?gene=EMP1) |  |  |
|  | superoxide dismutase 2, mitochondrial | [SOD2](http://www.genecards.org/cgi-bin/carddisp.pl?gene=SOD2) |  |  |
|  | transporter 2, ATP-binding cassette, sub-family B (MDR/TAP) | [TAP2](http://www.genecards.org/cgi-bin/carddisp.pl?gene=TAP2) |  |  |
|  | EPH receptor A2 | [EPHA2](http://www.genecards.org/cgi-bin/carddisp.pl?gene=EPHA2) |  |  |
|  | leucine rich repeat containing 32 | [LRRC32](http://www.genecards.org/cgi-bin/carddisp.pl?gene=LRRC32) |  |  |
|  | B-cell CLL/lymphoma 6 | [BCL6](http://www.genecards.org/cgi-bin/carddisp.pl?gene=BCL6) |  |  |
|  | B-cell CLL/lymphoma 6 | [BCL6](http://www.genecards.org/cgi-bin/carddisp.pl?gene=BCL6) |  |  |
|  | BCL2-associated athanogene 3 | [BAG3](http://www.genecards.org/cgi-bin/carddisp.pl?gene=BAG3) |  |  |
|  | nicotinamide N-methyltransferase | [NNMT](http://www.genecards.org/cgi-bin/carddisp.pl?gene=NNMT) |  |  |
|  | ceruloplasmin (ferroxidase) | [CP](http://www.genecards.org/cgi-bin/carddisp.pl?gene=CP) |  |  |
|  | serine/threonine kinase 17b | [STK17B](http://www.genecards.org/cgi-bin/carddisp.pl?gene=STK17B) |  |  |
|  | matrix metallopeptidase 19 | [MMP19](http://www.genecards.org/cgi-bin/carddisp.pl?gene=MMP19) |  |  |
|  | elongation factor, RNA polymerase II, 2 | [ELL2](http://www.genecards.org/cgi-bin/carddisp.pl?gene=ELL2) |  |  |
|  | CCAAT/enhancer binding protein (C/EBP), alpha | [CEBPA](http://www.genecards.org/cgi-bin/carddisp.pl?gene=CEBPA) |  |  |
|  | low density lipoprotein receptor | [LDLR](http://www.genecards.org/cgi-bin/carddisp.pl?gene=LDLR) |  |  |
|  | low density lipoprotein receptor | [LDLR](http://www.genecards.org/cgi-bin/carddisp.pl?gene=LDLR) |  |  |
|  | low density lipoprotein receptor | [LDLR](http://www.genecards.org/cgi-bin/carddisp.pl?gene=LDLR) |  |  |
|  | GTP cyclohydrolase 1 | [GCH1](http://www.genecards.org/cgi-bin/carddisp.pl?gene=GCH1) |  |  |
| **Monocyte 4 (3.1%) 0.013** | | | | |
|  | Fc fragment of IgA, receptor for | [FCAR](http://www.genecards.org/cgi-bin/carddisp.pl?gene=FCAR) |  |  |
|  | chemokine (C-C motif) ligand 23 | [CCL23](http://www.genecards.org/cgi-bin/carddisp.pl?gene=CCL23) |  |  |
|  | formyl peptide receptor 2 | [FPR2](http://www.genecards.org/cgi-bin/carddisp.pl?gene=FPR2) |  |  |
|  | formyl peptide receptor 2 | [FPR2](http://www.genecards.org/cgi-bin/carddisp.pl?gene=FPR2) |  |  |
|  | transmembrane 7 superfamily member 4 | [TM7SF4](http://www.genecards.org/cgi-bin/carddisp.pl?gene=TM7SF4) |  |  |
| **Skeletal muscle 11 (8.4%)**  **0.016** | | | | |
|  | microfibrillar associated protein 5 | [MFAP5](http://www.genecards.org/cgi-bin/carddisp.pl?gene=MFAP5) |  |  |
|  | spectrin repeat containing, nuclear envelope 1 | [SYNE1](http://www.genecards.org/cgi-bin/carddisp.pl?gene=SYNE1) |  |  |
|  | fatty acid binding protein 3, muscle and heart (mammary-derived growth inhibitor) | [FABP3](http://www.genecards.org/cgi-bin/carddisp.pl?gene=FABP3) |  |  |
|  | solute carrier family 19 (thiamine transporter), member 2 | [SLC19A2](http://www.genecards.org/cgi-bin/carddisp.pl?gene=SLC19A2) |  |  |
|  | kelch repeat and BTB (POZ) domain containing 10 | [KBTBD10](http://www.genecards.org/cgi-bin/carddisp.pl?gene=KBTBD10) |  |  |
|  | protein phosphatase 1, regulatory (inhibitor) subunit 3C | [PPP1R3C](http://www.genecards.org/cgi-bin/carddisp.pl?gene=PPP1R3C) |  |  |
|  | nuclear receptor subfamily 4, group A, member 3 | [NR4A3](http://www.genecards.org/cgi-bin/carddisp.pl?gene=NR4A3) |  |  |
|  | nuclear receptor subfamily 4, group A, member 3 | [NR4A3](http://www.genecards.org/cgi-bin/carddisp.pl?gene=NR4A3) |  |  |
|  | B-cell CLL/lymphoma 6 | [BCL6](http://www.genecards.org/cgi-bin/carddisp.pl?gene=BCL6) |  |  |
|  | B-cell CLL/lymphoma 6 | [BCL6](http://www.genecards.org/cgi-bin/carddisp.pl?gene=BCL6) |  |  |
|  | CD36 molecule (thrombospondin receptor) | [CD36](http://www.genecards.org/cgi-bin/carddisp.pl?gene=CD36) |  |  |
|  | 6-phosphofructo-2-kinase/fructose-2,6-biphosphatase 3 | [PFKFB3](http://www.genecards.org/cgi-bin/carddisp.pl?gene=PFKFB3) |  |  |
|  | myeloid leukemia factor 1 | [MLF1](http://www.genecards.org/cgi-bin/carddisp.pl?gene=MLF1) |  |  |
| **Endothelial cell 4 (3.1%) 0.017** | | | | |
|  | thrombospondin 1 | [THBS1](http://www.genecards.org/cgi-bin/carddisp.pl?gene=THBS1) |  |  |
|  | thrombospondin 1 | [THBS1](http://www.genecards.org/cgi-bin/carddisp.pl?gene=THBS1) |  |  |
|  | thrombospondin 1 | [THBS1](http://www.genecards.org/cgi-bin/carddisp.pl?gene=THBS1) |  |  |
|  | pentraxin-related gene, rapidly induced by IL-1 beta | [PTX3](http://www.genecards.org/cgi-bin/carddisp.pl?gene=PTX3) |  |  |
|  | ADAM metallopeptidase with thrombospondin type 1 motif, 1 | [ADAMTS1](http://www.genecards.org/cgi-bin/carddisp.pl?gene=ADAMTS1) |  |  |
|  | FOS-like antigen 2 | [FOSL2](http://www.genecards.org/cgi-bin/carddisp.pl?gene=FOSL2) |  |  |
|  | FOS-like antigen 2 | [FOSL2](http://www.genecards.org/cgi-bin/carddisp.pl?gene=FOSL2) |  |  |
|  | FOS-like antigen 2 | [FOSL2](http://www.genecards.org/cgi-bin/carddisp.pl?gene=FOSL2) |  |  |
| **Plasma 8 (6.1%) 0.019** | | | | |
|  | thrombospondin 1 | [THBS1](http://www.genecards.org/cgi-bin/carddisp.pl?gene=THBS1) |  |  |
|  | thrombospondin 1 | [THBS1](http://www.genecards.org/cgi-bin/carddisp.pl?gene=THBS1) |  |  |
|  | thrombospondin 1 | [THBS1](http://www.genecards.org/cgi-bin/carddisp.pl?gene=THBS1) |  |  |
|  | ceruloplasmin (ferroxidase) | [CP](http://www.genecards.org/cgi-bin/carddisp.pl?gene=CP) |  |  |
|  | neurotrophic tyrosine kinase, receptor, type 2 | [NTRK2](http://www.genecards.org/cgi-bin/carddisp.pl?gene=NTRK2) |  |  |
|  | coagulation factor VIII, procoagulant component | [F8](http://www.genecards.org/cgi-bin/carddisp.pl?gene=F8) |  |  |
|  | periostin, osteoblast specific factor | [POSTN](http://www.genecards.org/cgi-bin/carddisp.pl?gene=POSTN) |  |  |
|  | low density lipoprotein receptor | [LDLR](http://www.genecards.org/cgi-bin/carddisp.pl?gene=LDLR) |  |  |
|  | low density lipoprotein receptor | [LDLR](http://www.genecards.org/cgi-bin/carddisp.pl?gene=LDLR) |  |  |
|  | low density lipoprotein receptor | [LDLR](http://www.genecards.org/cgi-bin/carddisp.pl?gene=LDLR) |  |  |
|  | serpin peptidase inhibitor, clade A (alpha-1 antiproteinase, antitrypsin), member 3 | [SERPINA3](http://www.genecards.org/cgi-bin/carddisp.pl?gene=SERPINA3) |  |  |
|  | fibroblast growth factor receptor 1 | [RPS20P22](http://www.genecards.org/cgi-bin/carddisp.pl?gene=RPS20P22) |  |  |
|  | fibroblast growth factor receptor 1 | [FGFR1](http://www.genecards.org/cgi-bin/carddisp.pl?gene=FGFR1) |  |  |
| **Lung** | | | 30 (22.9%) | 0.039 |
|  | STEAP family member 4 | [STEAP4](http://www.genecards.org/cgi-bin/carddisp.pl?gene=STEAP4) |  |  |
|  | ubiquitin carboxyl-terminal esterase L1 (ubiquitin thiolesterase) | [UCHL1](http://www.genecards.org/cgi-bin/carddisp.pl?gene=UCHL1) |  |  |
|  | serine dehydratase | [SDS](http://www.genecards.org/cgi-bin/carddisp.pl?gene=SDS) |  |  |
|  | thyrotropin-releasing hormone degrading enzyme | [TRHDE](http://www.genecards.org/cgi-bin/carddisp.pl?gene=TRHDE) |  |  |
|  | defensin, alpha 1 | [DEFA1](http://www.genecards.org/cgi-bin/carddisp.pl?gene=DEFA1) |  |  |
|  | fibroblast growth factor receptor 1 | [FGFR1](http://www.genecards.org/cgi-bin/carddisp.pl?gene=FGFR1) |  |  |
|  | fibroblast growth factor receptor 1 | [FGFR1](http://www.genecards.org/cgi-bin/carddisp.pl?gene=FGFR1) |  |  |
|  | suppressor of cytokine signaling 2 | [SOCS2](http://www.genecards.org/cgi-bin/carddisp.pl?gene=SOCS2) |  |  |
|  | suppressor of cytokine signaling 2 | [SOCS2](http://www.genecards.org/cgi-bin/carddisp.pl?gene=SOCS2) |  |  |
|  | E74-like factor 5 (ets domain transcription factor) | [ELF5](http://www.genecards.org/cgi-bin/carddisp.pl?gene=ELF5) |  |  |
|  | serpin peptidase inhibitor, clade E (nexin, plasminogen activator inhibitor type 1), member 1 | [SERPINE1](http://www.genecards.org/cgi-bin/carddisp.pl?gene=SERPINE1) |  |  |
|  | hemoglobin, gamma A | [HBG1](http://www.genecards.org/cgi-bin/carddisp.pl?gene=HBG1) |  |  |
|  | coagulation factor III (thromboplastin, tissue factor) | [F3](http://www.genecards.org/cgi-bin/carddisp.pl?gene=F3) |  |  |
|  | arginase, type II | [ARG2](http://www.genecards.org/cgi-bin/carddisp.pl?gene=ARG2) |  |  |
|  | Krüppel-like factor 4 (gut) | [KLF4](http://www.genecards.org/cgi-bin/carddisp.pl?gene=KLF4) |  |  |
|  | epithelial membrane protein 1 | [EMP1](http://www.genecards.org/cgi-bin/carddisp.pl?gene=EMP1) |  |  |
|  | epithelial membrane protein 1 | [EMP1](http://www.genecards.org/cgi-bin/carddisp.pl?gene=EMP1) |  |  |
|  | epithelial membrane protein 1 | [Hs.436298](http://www.ncbi.nlm.nih.gov/UniGene/clust.cgi?ORG=Hs&CID=436298) |  |  |
|  | carbonic anhydrase XII | [CA12](http://www.genecards.org/cgi-bin/carddisp.pl?gene=CA12) |  |  |
|  | carbonic anhydrase XII | [CA12](http://www.genecards.org/cgi-bin/carddisp.pl?gene=CA12) |  |  |
|  | carbonic anhydrase XII | [CA12](http://www.genecards.org/cgi-bin/carddisp.pl?gene=CA12) |  |  |
|  | immunoglobulin superfamily, member 6 | [IGSF6](http://www.genecards.org/cgi-bin/carddisp.pl?gene=IGSF6) |  |  |
|  | achaete-scute complex homolog 1 (Drosophila) | [ASCL1](http://www.genecards.org/cgi-bin/carddisp.pl?gene=ASCL1) |  |  |
|  | superoxide dismutase 2, mitochondrial | [SOD2](http://www.genecards.org/cgi-bin/carddisp.pl?gene=SOD2) |  |  |
|  | E74-like factor 3 (ets domain transcription factor, epithelial-specific ) | [ELF3](http://www.genecards.org/cgi-bin/carddisp.pl?gene=ELF3) |  |  |
|  | heat shock 70kDa protein 2 | [HSPA2](http://www.genecards.org/cgi-bin/carddisp.pl?gene=HSPA2) |  |  |
|  | tripartite motif-containing 58 | [TRIM58](http://www.genecards.org/cgi-bin/carddisp.pl?gene=TRIM58) |  |  |
|  | neural precursor cell expressed, developmentally down-regulated 9 | [NEDD9](http://www.genecards.org/cgi-bin/carddisp.pl?gene=NEDD9) |  |  |
|  | cytochrome P450, family 1, subfamily B, polypeptide 1 | [CYP1B1](http://www.genecards.org/cgi-bin/carddisp.pl?gene=CYP1B1) |  |  |
|  | cytochrome P450, family 1, subfamily B, polypeptide 1 | [CYP1B1](http://www.genecards.org/cgi-bin/carddisp.pl?gene=CYP1B1) |  |  |
|  | nicotinamide phosphoribosyltransferase | [NAMPT](http://www.genecards.org/cgi-bin/carddisp.pl?gene=NAMPT) |  |  |
|  | nicotinamide phosphoribosyltransferase | [Hs.489615](http://www.ncbi.nlm.nih.gov/UniGene/clust.cgi?ORG=Hs&CID=489615) |  |  |
|  | Krüppel-like factor 9 | [KLF9](http://www.genecards.org/cgi-bin/carddisp.pl?gene=KLF9) |  |  |
|  | Krüppel-like factor 9 | [KLF9](http://www.genecards.org/cgi-bin/carddisp.pl?gene=KLF9) |  |  |
|  | BCL2-associated athanogene 3 | [BAG3](http://www.genecards.org/cgi-bin/carddisp.pl?gene=BAG3) |  |  |
|  | FOS-like antigen 2 | [FOSL2](http://www.genecards.org/cgi-bin/carddisp.pl?gene=FOSL2) |  |  |
|  | FOS-like antigen 2 | [FOSL2](http://www.genecards.org/cgi-bin/carddisp.pl?gene=FOSL2) |  |  |
|  | FOS-like antigen 2 | [FOSL2](http://www.genecards.org/cgi-bin/carddisp.pl?gene=FOSL2) |  |  |
|  | thrombomodulin | [THBD](http://www.genecards.org/cgi-bin/carddisp.pl?gene=THBD) |  |  |
|  | thrombomodulin | [THBD](http://www.genecards.org/cgi-bin/carddisp.pl?gene=THBD) |  |  |
|  | deoxyribonuclease II beta | [DNASE2B](http://www.genecards.org/cgi-bin/carddisp.pl?gene=DNASE2B) |  |  |
|  | cold shock domain protein A; cold shock domain protein A pseudogene 1 | [CSDA](http://www.genecards.org/cgi-bin/carddisp.pl?gene=CSDA) |  |  |
| **Granulocyte 2 (1.5%) 0.0416** | | | | |
|  | formyl peptide receptor 2 | [FPR2](http://www.genecards.org/cgi-bin/carddisp.pl?gene=FPR2) |  |  |
|  | formyl peptide receptor 2 | [FPR2](http://www.genecards.org/cgi-bin/carddisp.pl?gene=FPR2) |  |  |
|  | GTP cyclohydrolase 1 | [GCH1](http://www.genecards.org/cgi-bin/carddisp.pl?gene=GCH1) |  |  |
