# Supplementary material for: Krüppel-like zinc finger proteins in end-stage COPD lungs with and without severe alpha1-antitrypsin deficiency
Source: Orphanet J Rare Dis. 2012 May 23;7:29. doi: 10.1186/1750-1172-7-29 (PMC3517304; doi:10.1186/1750-1172-7-29)
Supplement: Additional file 3 — Table S3. Disease-associated genes enriched in end-stage COPD lung tissue from ZZ compared to MM AAT patients. [file 1750-1172-7-29-S3.doc]

**Supplement Table 3**. Disease-associated genes enriched in end-stage COPD lung tissue from ZZ compared to MM AAT patients.

| **Term** | **Gene name** | **Gene Symbol** | **Gene number (%)** | **P value** |
| --- | --- | --- | --- | --- |
| **Diabetes type 2** | | | **11 (8.4%)** | **0.013** |
|  | serpin peptidase inhibitor, clade E (nexin, plasminogen activator inhibitor type 1), member 1 | [SERPINE1](http://www.genecards.org/cgi-bin/carddisp.pl?gene=SERPINE1) |  |  |
|  | hematopoietically expressed homeobox | [HHEX](http://www.genecards.org/cgi-bin/carddisp.pl?gene=HHEX) |  |  |
|  | hematopoietically expressed homeobox | [HHEX](http://www.genecards.org/cgi-bin/carddisp.pl?gene=HHEX) |  |  |
|  | low density lipoprotein receptor | [LDLR](http://www.genecards.org/cgi-bin/carddisp.pl?gene=LDLR) |  |  |
|  | low density lipoprotein receptor | [LDLR](http://www.genecards.org/cgi-bin/carddisp.pl?gene=LDLR) |  |  |
|  | low density lipoprotein receptor | [LDLR](http://www.genecards.org/cgi-bin/carddisp.pl?gene=LDLR) |  |  |
|  | fatty acid binding protein 3, muscle and heart (mammary-derived growth inhibitor) | [FABP3](http://www.genecards.org/cgi-bin/carddisp.pl?gene=FABP3) |  |  |
|  | solute carrier family 19 (thiamine transporter), member 2 | [SLC19A2](http://www.genecards.org/cgi-bin/carddisp.pl?gene=SLC19A2) |  |  |
|  | coagulation factor III (thromboplastin, tissue factor) | [F3](http://www.genecards.org/cgi-bin/carddisp.pl?gene=F3) |  |  |
|  | Krüppel-like factor 4 (gut) | [KLF4](http://www.genecards.org/cgi-bin/carddisp.pl?gene=KLF4) |  |  |
|  | CD36 molecule (thrombospondin receptor) | [CD36](http://www.genecards.org/cgi-bin/carddisp.pl?gene=CD36) |  |  |
|  | nicotinamide phosphoribosyltransferase | [NAMPT](http://www.genecards.org/cgi-bin/carddisp.pl?gene=NAMPT) |  |  |
|  | nicotinamide phosphoribosyltransferase | [Hs.489615](http://www.ncbi.nlm.nih.gov/UniGene/clust.cgi?ORG=Hs&CID=489615) |  |  |
|  | superoxide dismutase 2, mitochondrial | [SOD2](http://www.genecards.org/cgi-bin/carddisp.pl?gene=SOD2) |  |  |
|  | suppressor of cytokine signaling 2 | [SOCS2](http://www.genecards.org/cgi-bin/carddisp.pl?gene=SOCS2) |  |  |
|  | suppressor of cytokine signaling 2 | [SOCS2](http://www.genecards.org/cgi-bin/carddisp.pl?gene=SOCS2) |  |  |
|  |  |  |  |  |
| **Parkinson’s disease** | | | **7(5.3%)** | **0.015** |
|  | ceruloplasmin (ferroxidase) | [CP](http://www.genecards.org/cgi-bin/carddisp.pl?gene=CP) |  |  |
|  | ubiquitin carboxyl-terminal esterase L1 (ubiquitin thiolesterase) | [UCHL1](http://www.genecards.org/cgi-bin/carddisp.pl?gene=UCHL1) |  |  |
|  | cytochrome P450, family 1, subfamily B, polypeptide 1 | [CYP1B1](http://www.genecards.org/cgi-bin/carddisp.pl?gene=CYP1B1) |  |  |
|  | cytochrome P450, family 1, subfamily B, polypeptide 1 | [CYP1B1](http://www.genecards.org/cgi-bin/carddisp.pl?gene=CYP1B1) |  |  |
|  | GTP cyclohydrolase 1 | [GCH1](http://www.genecards.org/cgi-bin/carddisp.pl?gene=GCH1) |  |  |
|  | serpin peptidase inhibitor, clade A (alpha-1 antiproteinase, antitrypsin), member 3 | [SERPINA3](http://www.genecards.org/cgi-bin/carddisp.pl?gene=SERPINA3) |  |  |
|  | superoxide dismutase 2, mitochondrial | [SOD2](http://www.genecards.org/cgi-bin/carddisp.pl?gene=SOD2) |  |  |
|  | achaete-scute complex homolog 1 (Drosophila) | [ASCL1](http://www.genecards.org/cgi-bin/carddisp.pl?gene=ASCL1) |  |  |
| **Alzheimer`s disease** | | | **9(6.9%)** | **0.033** |
|  | ubiquitin carboxyl-terminal esterase L1 (ubiquitin thiolesterase) | [UCHL1](http://www.genecards.org/cgi-bin/carddisp.pl?gene=UCHL1) |  |  |
|  | serpin peptidase inhibitor, clade E (nexin, plasminogen activator inhibitor type 1), member 1 | [SERPINE1](http://www.genecards.org/cgi-bin/carddisp.pl?gene=SERPINE1) |  |  |
|  | hematopoietically expressed homeobox | [HHEX](http://www.genecards.org/cgi-bin/carddisp.pl?gene=HHEX) |  |  |
|  | hematopoietically expressed homeobox | [HHEX](http://www.genecards.org/cgi-bin/carddisp.pl?gene=HHEX) |  |  |
|  | low density lipoprotein receptor | [LDLR](http://www.genecards.org/cgi-bin/carddisp.pl?gene=LDLR) |  |  |
|  | low density lipoprotein receptor | [LDLR](http://www.genecards.org/cgi-bin/carddisp.pl?gene=LDLR) |  |  |
|  | low density lipoprotein receptor | [LDLR](http://www.genecards.org/cgi-bin/carddisp.pl?gene=LDLR) |  |  |
|  | 3-hydroxy-3-methylglutaryl-Coenzyme A synthase 2 (mitochondrial) | [HMGCS2](http://www.genecards.org/cgi-bin/carddisp.pl?gene=HMGCS2) |  |  |
|  | serpin peptidase inhibitor, clade A (alpha-1 antiproteinase, antitrypsin), member 3 | [SERPINA3](http://www.genecards.org/cgi-bin/carddisp.pl?gene=SERPINA3) |  |  |
|  | superoxide dismutase 2, mitochondrial | [SOD2](http://www.genecards.org/cgi-bin/carddisp.pl?gene=SOD2) |  |  |
|  | transporter 2, ATP-binding cassette, sub-family B (MDR/TAP) | [TAP2](http://www.genecards.org/cgi-bin/carddisp.pl?gene=TAP2) |  |  |
|  | heat shock 70kDa protein 2 | [HSPA2](http://www.genecards.org/cgi-bin/carddisp.pl?gene=HSPA2) |  |  |
| **Polyneuropathy vasculitis** | | | **2 (1.2%)** | **0.033** |
|  | CD1a molecule | [CD1A](http://www.genecards.org/cgi-bin/carddisp.pl?gene=CD1A) |  |  |
|  | CD1e molecule | [CD1E](http://www.genecards.org/cgi-bin/carddisp.pl?gene=CD1E) |  |  |
