# Supplementary material for: Krüppel-like zinc finger proteins in end-stage COPD lungs with and without severe alpha1-antitrypsin deficiency
Source: Orphanet J Rare Dis. 2012 May 23;7:29. doi: 10.1186/1750-1172-7-29 (PMC3517304; doi:10.1186/1750-1172-7-29)
Supplement: Additional file 4 — Table S4. More highly expressed genes associated with biological processes in end-stage COPD lung tissue from ZZ compared to MM AAT patients. [file 1750-1172-7-29-S4.doc]

**Supplement Table 4**. More highly expressed genes associated with biological processes in end-stage COPD lung tissue from ZZ compared to MM AAT patients.

| **Term** | **Gene name** | **Gene Symbol** |
| --- | --- | --- |
| **GO:0010033~response to organic substance** | | |
|  | retinol binding protein 4, plasma | [**RBP4**](http://www.genecards.org/cgi-bin/carddisp.pl?gene=RBP4) |
|  | fatty acid binding protein 3, muscle and heart (mammary-derived growth inhibitor) | [**FABP3**](http://www.genecards.org/cgi-bin/carddisp.pl?gene=FABP3) |
|  | cytochrome P450, family 1, subfamily B, polypeptide 1 | [**CYP1B1**](http://www.genecards.org/cgi-bin/carddisp.pl?gene=CYP1B1) |
|  | cytochrome P450, family 1, subfamily B, polypeptide 1 | [**CYP1B1**](http://www.genecards.org/cgi-bin/carddisp.pl?gene=CYP1B1) |
|  | potassium large conductance calcium-activated channel, subfamily M, alpha member 1 | [**KCNMA1**](http://www.genecards.org/cgi-bin/carddisp.pl?gene=KCNMA1) |
|  | nuclear receptor subfamily 4, group A, member 3 | [**NR4A3**](http://www.genecards.org/cgi-bin/carddisp.pl?gene=NR4A3) |
|  | nuclear receptor subfamily 4, group A, member 3 | [**NR4A3**](http://www.genecards.org/cgi-bin/carddisp.pl?gene=NR4A3) |
|  | phosphoinositide-3-kinase, regulatory subunit 3 (gamma) | [**PIK3R3**](http://www.genecards.org/cgi-bin/carddisp.pl?gene=PIK3R3) |
|  | msh homeobox 1 | [**MSX1**](http://www.genecards.org/cgi-bin/carddisp.pl?gene=MSX1) |
|  | suppressor of cytokine signaling 2 | [**SOCS2**](http://www.genecards.org/cgi-bin/carddisp.pl?gene=SOCS2) |
|  | suppressor of cytokine signaling 2 | [**SOCS2**](http://www.genecards.org/cgi-bin/carddisp.pl?gene=SOCS2) |
|  | thrombospondin 1 | [**THBS1**](http://www.genecards.org/cgi-bin/carddisp.pl?gene=THBS1) |
|  | thrombospondin 1 | [**THBS1**](http://www.genecards.org/cgi-bin/carddisp.pl?gene=THBS1) |
|  | thrombospondin 1 | [**THBS1**](http://www.genecards.org/cgi-bin/carddisp.pl?gene=THBS1) |
|  | Thrombomodulin | [**THBD**](http://www.genecards.org/cgi-bin/carddisp.pl?gene=THBD) |
|  | Thrombomodulin | [**THBD**](http://www.genecards.org/cgi-bin/carddisp.pl?gene=THBD) |
|  | myeloid cell leukemia sequence 1 (BCL2-related) | [**Hs.723145**](http://www.ncbi.nlm.nih.gov/UniGene/clust.cgi?ORG=Hs&CID=723145) |
|  | myeloid cell leukemia sequence 1 (BCL2-related) | **MCL1** |
|  | myeloid cell leukemia sequence 1 (BCL2-related) | [**MCL1**](http://www.genecards.org/cgi-bin/carddisp.pl?gene=MCL1) |
|  | low density lipoprotein receptor | [**LDLR**](http://www.genecards.org/cgi-bin/carddisp.pl?gene=LDLR) |
|  | low density lipoprotein receptor | [**LDLR**](http://www.genecards.org/cgi-bin/carddisp.pl?gene=LDLR) |
|  | low density lipoprotein receptor | [**LDLR**](http://www.genecards.org/cgi-bin/carddisp.pl?gene=LDLR) |
|  | 3-hydroxy-3-methylglutaryl-coenzyme A synthase 2 (mitochondrial) | [**HMGCS2**](http://www.genecards.org/cgi-bin/carddisp.pl?gene=HMGCS2) |
|  | GTP cyclohydrolase 1 | [**GCH1**](http://www.genecards.org/cgi-bin/carddisp.pl?gene=GCH1) |
|  | oncostatin M receptor | [**OSMR**](http://www.genecards.org/cgi-bin/carddisp.pl?gene=OSMR) |
|  | Pannexin 1 | **PANX 1** |
|  | Krüppel-like factor 10 | [**KLF10**](http://www.genecards.org/cgi-bin/carddisp.pl?gene=KLF10) |
|  | transporter 2, ATP-binding cassette, sub-family B (MDR/TAP) | [**TAP2**](http://www.genecards.org/cgi-bin/carddisp.pl?gene=TAP2) |
|  | heat shock 70kDa protein 2 | [**HSPA2**](http://www.genecards.org/cgi-bin/carddisp.pl?gene=HSPA2) |
| **GO:0042127~regulation of cell proliferation** | | |
|  | retinol binding protein 4, plasma | [**RBP4**](http://www.genecards.org/cgi-bin/carddisp.pl?gene=RBP4) |
|  | fatty acid binding protein 3, muscle and heart (mammary-derived growth inhibitor) | [**FABP3**](http://www.genecards.org/cgi-bin/carddisp.pl?gene=FABP3) |
|  | B-cell CLL/lymphoma 6 | [**BCL6**](http://www.genecards.org/cgi-bin/carddisp.pl?gene=BCL6) |
|  | B-cell CLL/lymphoma 6 | [**BCL6**](http://www.genecards.org/cgi-bin/carddisp.pl?gene=BCL6) |
|  | nicotinamide phosphoribosyltransferase | [**NAMPT**](http://www.genecards.org/cgi-bin/carddisp.pl?gene=NAMPT) |
|  | nicotinamide phosphoribosyltransferase | [**Hs.489615**](http://www.ncbi.nlm.nih.gov/UniGene/clust.cgi?ORG=Hs&CID=489615) |
|  | fibroblast growth factor receptor 1 | [**FGFR1**](http://www.genecards.org/cgi-bin/carddisp.pl?gene=FGFR1) |
|  | fibroblast growth factor receptor 1 | [**RPS20P22**](http://www.genecards.org/cgi-bin/carddisp.pl?gene=RPS20P22) |
|  | fms-related tyrosine kinase 1 (vascular endothelial growth factor/vascular permeability factor receptor) | [**FLT1**](http://www.genecards.org/cgi-bin/carddisp.pl?gene=FLT1) |
|  | msh homeobox 1 | [**MSX1**](http://www.genecards.org/cgi-bin/carddisp.pl?gene=MSX1) |
|  | FOS-like antigen 2 | [**FOSL2**](http://www.genecards.org/cgi-bin/carddisp.pl?gene=FOSL2) |
|  | FOS-like antigen 2 | [**FOSL2**](http://www.genecards.org/cgi-bin/carddisp.pl?gene=FOSL2) |
|  | FOS-like antigen 2 | [**FOSL2**](http://www.genecards.org/cgi-bin/carddisp.pl?gene=FOSL2) |
|  | thrombospondin 1 | [**THBS1**](http://www.genecards.org/cgi-bin/carddisp.pl?gene=THBS1) |
|  | thrombospondin 1 | [**THBS1**](http://www.genecards.org/cgi-bin/carddisp.pl?gene=THBS1) |
|  | thrombospondin 1 | [**THBS1**](http://www.genecards.org/cgi-bin/carddisp.pl?gene=THBS1) |
|  | Epiregulin | [**EREG**](http://www.genecards.org/cgi-bin/carddisp.pl?gene=EREG) |
|  | ADAM metallopeptidase with thrombospondin type 1 motif, 1 | [**ADAMTS1**](http://www.genecards.org/cgi-bin/carddisp.pl?gene=ADAMTS1) |
|  | CCAAT/enhancer binding protein (C/EBP), alpha | [**CEBPA**](http://www.genecards.org/cgi-bin/carddisp.pl?gene=CEBPA) |
|  | serpin peptidase inhibitor, clade E (nexin, plasminogen activator inhibitor type 1), member 1 | [**SERPINE1**](http://www.genecards.org/cgi-bin/carddisp.pl?gene=SERPINE1) |
|  | hematopoietically expressed homeobox | [**HHEX**](http://www.genecards.org/cgi-bin/carddisp.pl?gene=HHEX) |
|  | hematopoietically expressed homeobox | [**HHEX**](http://www.genecards.org/cgi-bin/carddisp.pl?gene=HHEX) |
|  | chemokine (C-C motif) ligand 23 | [**CCL23**](http://www.genecards.org/cgi-bin/carddisp.pl?gene=CCL23) |
|  | coagulation factor III (thromboplastin, tissue factor) | [**F3**](http://www.genecards.org/cgi-bin/carddisp.pl?gene=F3) |
|  | Krüppel-like factor 4 (gut) | [**KLF4**](http://www.genecards.org/cgi-bin/carddisp.pl?gene=KLF4) |
|  | oncostatin M receptor | [**OSMR**](http://www.genecards.org/cgi-bin/carddisp.pl?gene=OSMR) |
|  | Krüppel-like factor 10 | [**KLF10**](http://www.genecards.org/cgi-bin/carddisp.pl?gene=KLF10) |
|  | superoxide dismutase 2, mitochondrial | [**SOD2**](http://www.genecards.org/cgi-bin/carddisp.pl?gene=SOD2) |
| **GO:0007169~transmembrane receptor protein tyrosine kinase signaling pathway** | | |
|  | TCDD-inducible poly(ADP-ribose) polymerase | [**TIPARP**](http://www.genecards.org/cgi-bin/carddisp.pl?gene=TIPARP) |
|  | amphiregulin; amphiregulin B | [**AREG**](http://www.genecards.org/cgi-bin/carddisp.pl?gene=AREG) |
|  | neurotrophic tyrosine kinase, receptor, type 2 | [**NTRK2**](http://www.genecards.org/cgi-bin/carddisp.pl?gene=NTRK2) |
|  | Epiregulin | [**EREG**](http://www.genecards.org/cgi-bin/carddisp.pl?gene=EREG) |
|  | AT rich interactive domain 5B (MRF1-like) | [**ARID5B**](http://www.genecards.org/cgi-bin/carddisp.pl?gene=ARID5B) |
|  | EPH receptor A2 | [**EPHA2**](http://www.genecards.org/cgi-bin/carddisp.pl?gene=EPHA2) |
|  | fibroblast growth factor receptor 1 | [**FGFR1**](http://www.genecards.org/cgi-bin/carddisp.pl?gene=FGFR1) |
|  | fibroblast growth factor receptor 1 | [**RPS20P22**](http://www.genecards.org/cgi-bin/carddisp.pl?gene=RPS20P22) |
|  | phosphoinositide-3-kinase, regulatory subunit 3 (gamma) | [**PIK3R3**](http://www.genecards.org/cgi-bin/carddisp.pl?gene=PIK3R3) |
|  | fms-related tyrosine kinase 1 (vascular endothelial growth factor/vascular permeability factor receptor) | [**FLT1**](http://www.genecards.org/cgi-bin/carddisp.pl?gene=FLT1) |
|  | suppressor of cytokine signaling 2 | [**SOCS2**](http://www.genecards.org/cgi-bin/carddisp.pl?gene=SOCS2) |
|  | suppressor of cytokine signaling 2 | [**SOCS2**](http://www.genecards.org/cgi-bin/carddisp.pl?gene=SOCS2) |
| **GO:0009611~response to injury** | | |
|  | coagulation factor VIII, procoagulant component | [**F8**](http://www.genecards.org/cgi-bin/carddisp.pl?gene=F8) |
|  | platelet factor 4 | [**PF4**](http://www.genecards.org/cgi-bin/carddisp.pl?gene=PF4) |
|  | formyl peptide receptor 2 | [**FPR2**](http://www.genecards.org/cgi-bin/carddisp.pl?gene=FPR2) |
|  | formyl peptide receptor 2 | [**FPR2**](http://www.genecards.org/cgi-bin/carddisp.pl?gene=FPR2) |
|  | CD36 molecule (thrombospondin receptor) | [**CD36**](http://www.genecards.org/cgi-bin/carddisp.pl?gene=CD36) |
|  | thrombospondin 1 | [**THBS1**](http://www.genecards.org/cgi-bin/carddisp.pl?gene=THBS1) |
|  | thrombospondin 1 | [**THBS1**](http://www.genecards.org/cgi-bin/carddisp.pl?gene=THBS1) |
|  | thrombospondin 1 | [**THBS1**](http://www.genecards.org/cgi-bin/carddisp.pl?gene=THBS1) |
|  | Thrombomodulin | [**THBD**](http://www.genecards.org/cgi-bin/carddisp.pl?gene=THBD) |
|  | Thrombomodulin | [**THBD**](http://www.genecards.org/cgi-bin/carddisp.pl?gene=THBD) |
|  | Epiregulin | [**EREG**](http://www.genecards.org/cgi-bin/carddisp.pl?gene=EREG) |
|  | pentraxin-related gene, rapidly induced by IL-1 beta | [**PTX3**](http://www.genecards.org/cgi-bin/carddisp.pl?gene=PTX3) |
|  | serpin peptidase inhibitor, clade E (nexin, plasminogen activator inhibitor type 1), member 1 | [**SERPINE1**](http://www.genecards.org/cgi-bin/carddisp.pl?gene=SERPINE1) |
|  | chemokine (C-C motif) ligand 23 | [**CCL23**](http://www.genecards.org/cgi-bin/carddisp.pl?gene=CCL23) |
|  | coagulation factor III (thromboplastin, tissue factor) | [**F3**](http://www.genecards.org/cgi-bin/carddisp.pl?gene=F3) |
|  | serpin peptidase inhibitor, clade A (alpha-1 antiproteinase, antitrypsin), member 3 | [**SERPINA3**](http://www.genecards.org/cgi-bin/carddisp.pl?gene=SERPINA3) |
|  | S100 calcium binding protein A12 | [**S100A12**](http://www.genecards.org/cgi-bin/carddisp.pl?gene=S100A12) |
|  | E74-like factor 3 (ets domain transcription factor, epithelial-specific ) | [**ELF3**](http://www.genecards.org/cgi-bin/carddisp.pl?gene=ELF3) |
|  | superoxide dismutase 2, mitochondrial | [**SOD2**](http://www.genecards.org/cgi-bin/carddisp.pl?gene=SOD2) |
| **GO:0007167~enzyme linked receptor protein signaling pathway** | | |
|  | TCDD-inducible poly(ADP-ribose) polymerase | **TIPARP** |
|  | amphiregulin; amphiregulin B | **AREG** |
|  | neurotrophic tyrosine kinase, receptor, type 2 | **NTRK2** |
|  | Epiregulin | **EREG** |
|  | EPH receptor A2 | **EPHA2** |
|  | AT rich interactive domain 5B (MRF1-like) | **ARID5B** |
|  | Krüppel-like factor 10 | **KLF10** |
|  | fibroblast growth factor receptor 1 | **RPS20P22** |
|  | fibroblast growth factor receptor 1 | **FGFR1** |
|  | phosphoinositide-3-kinase, regulatory subunit 3 (gamma) | **PIK3R3** |
|  | fms-related tyrosine kinase 1 (vascular endothelial growth factor/vascular permeability factor receptor) | **FLT1** |
|  | msh homeobox 1 | **MSX1** |
|  | suppressor of cytokine signaling 2 | **SOCS2** |
